# Longitudinal Surveillance of Invasive Mosquitoes at Points of Entry in Northeastern Italy

**DOI:** 10.3390/tropicalmed11090247

**Published:** 2026-08-31

**Authors:** Sara Manzi, Martina Micocci, Luca Tassoni, Stefano Vettore, Davide Bonetto, Simone Martini, Francesco Gradoni, Federica Toniolo, Giulia Iandiorio, Lidia Iustina Danca, Giulia Chiarello, Patrizia Visentin, Matteo Cecconello, Sara Carlin, Federica Gobbo, Matteo Mazzucato, Francesca Russo, Chiara Ziprani, Barbra Bucci, Sergio Iavicoli, Vincenzo Severino, Fabrizio Montarsi

**Affiliations:** 1 Istituto Zooprofilattico Sperimentale delle Venezie, 35020 Legnaro, PD, Italy; ltassoni@izsvenezie.it (L.T.); fgradoni@izsvenezie.it (F.G.); ftoniolo@izsvenezie.it (F.T.); giandiorio@izsvenezie.it (G.I.); ldanca@izsvenezie.it (L.I.D.); gchiarello@izsvenezie.it (G.C.); fgobbo@izsvenezie.it (F.G.); mmazzucato@izsvenezie.it (M.M.); fmontarsi@izsvenezie.it (F.M.); 2 Entostudio S.r.l., 35020 Ponte San Nicolò, PD, Italy; 3 Prevention, Food Safety and Veterinary, Veneto Region, 30123 Venice, VE, Italy; 4 Ministry of Health, General Direction of Prevention, 00144 Rome, RM, Italyb.bucci@sanita.it (B.B.);; 5 Department of Prevention, Azienda ULSS 3 Serenissima, 30174 Venice, VE, Italy

**Keywords:** *Aedes* invasive mosquitoes, Points of Entry, surveillance, *Aedes koreicus*, *Aedes albopictus*

## Abstract

*Aedes* invasive mosquito (AIM) species are expanding worldwide, increasing the risk of arbovirus introduction and transmission. Points of Entry (PoEs), including airports and seaports, represent critical gateways for the introduction and spread of invasive vectors through international passenger and cargo traffic. This study evaluated the AIM long-term dynamics at three major PoEs (Treviso Airport, Venice Airport, and Venice Port) in northeastern Italy (Veneto region) between 2018 and 2024 through an integrated entomological surveillance programme designed to collect AIMs (adults and eggs) throughout the seasonal activity (June–October). Mosquitoes were identified morphologically and, where required, by molecular analyses. *Aedes albopictus* was the dominant AIM throughout the study period (eggs N = 96,192, adults N = 14,231), being detected at all three study sites and showing consistent interannual and seasonal dynamics, with peak abundance during summer despite routine vector control interventions. *Aedes koreicus* was detected at two of the three study sites, namely Venice and Treviso airports (eggs N = 1, adults N = 3). Phylogenetic analyses based on COI indicated that these specimens belonged to a distinct European lineage, revealing genetic diversity among European populations of *Ae. koreicus*. No evidence of newly established AIMs was found during the surveillance period. These findings highlight the importance of long-term surveillance at PoEs for the early detection of AIMs, monitoring temporal changes in established vector populations, and supporting integrated control and surveillance strategies to reduce the risk of vector establishment and mosquito-borne disease transmission.

## 1. Introduction

Mosquito-borne diseases represent one of the most rapidly expanding groups of infectious diseases worldwide, and their epidemiology has undergone substantial changes in recent decades [1,2]. Globalization, climate change, urbanization and increasing human mobility have facilitated both the spread of mosquito vectors and the subsequent introduction of arboviruses into previously unaffected regions [3,4]. In particular, human-mediated transportation—intensified by international travel and trade—has enabled the passive transport of mosquitoes and their eggs through vehicles, aircraft, ships and transported goods. Mosquito eggs and larvae can survive long-distance transport in artificial containers such as used tyres, ornamental plants, and cargo materials, while adults may be carried within vehicles or aircraft cabins [5,6,7,8]. Together, these processes have contributed to the expansion of *Aedes* invasive mosquito (AIM) species beyond their native ranges, altering the spatial dynamics of vector-borne diseases [9,10].

Among AIMs, those belonging to the genus *Aedes* represent a major public health concern. Species such as *Aedes albopictus* (Skuse, 1894), *Ae. aegypti* (Linnaeus, 1762), *Ae. japonicus* (Theobald, 1901) and *Ae. koreicus* (Edwards, 1917) have demonstrated remarkable ecological plasticity and the ability to rapidly colonise new environments in temperate regions [11,12,13,14,15]. These mosquitoes are able to exploit artificial breeding sites commonly found in urban and peri-urban environments, including containers, discarded tyres, flowerpots and rainwater collection systems. Their ecological adaptability, combined with the capacity of their eggs to survive adverse environmental conditions through diapause, has facilitated their successful establishment across many European countries [16]. The expansion of invasive *Aedes* species is particularly relevant from a public health perspective because several of these mosquitoes are competent vectors of arboviruses of global importance, including dengue (DENV), chikungunya (CHIKV), Zika (ZIKV) and West Nile viruses [17,18,19,20]. In recent years, the global incidence of mosquito-borne diseases has increased significantly, with DENV, CHIKV and ZIKV expanding beyond their traditional endemic regions [1,2]. In Europe, the establishment of *Ae. albopictus* has already enabled local transmission of tropical dengue and chikungunya fever, with the largest outbreak having occurred in Italy [21,22,23,24,25,26,27]. These episodes demonstrate that once competent vectors become established in a new area, imported cases associated with infected travellers may trigger local transmission cycles under favourable environmental conditions.

Within this context, Points of Entry (PoEs)—defined by the International Health Regulations (IHR, 2005) [28] as passages for international entry or exit of travellers, baggage, cargo, containers, conveyances, goods and postal parcels, including airports, seaports and major transportation hubs—represent critical gateways for the introduction and spread of vectors and associated pathogens. The importance of monitoring these locations is recognised within the framework of the IHR (2005) [28]. Under the IHR (2005), the World Health Organization (WHO) developed specific guidance for Member States on systematic vector surveillance and control at ports, airports, and ground crossings [29]. The WHO framework recommends establishing surveillance plans targeting both immature stages (e.g., eggs and larvae) and adult vectors, and implementing integrated vector management (IVM) to prevent the establishment and spread of invasive vectors and their international dissemination via land, air, and sea transport [29]. Under Annex 5 of the IHR (2005), vector control programmes should extend to a minimum 400-metre perimeter around PoE operational areas, with extension of this perimeter when vectors with a greater range are present. This perimeter may therefore be adapted according to local risk, vector flight range, and the characteristics of the PoE and its surrounding area [28]. In order to support and harmonize surveillance activities in Europe, the European Centre for Disease Prevention and Control (ECDC) has also developed operational guidance highlighting the importance of systematic vector surveillance at PoEs as part of IVM strategies [30]. Specifically, recommended surveillance activities include the use of ovitraps, adult mosquito traps and routine inspections in areas with a high probability of vector introduction, such as cargo handling zones, warehouses, aircraft unloading areas and container storage facilities [30,31].

Italy represents one of the European countries most affected by AIMs. The introduction of *Ae. albopictus* in the early 1990s [32], followed by its rapid spread and establishment across the country, and the discovery of additional AIMs in subsequent years, particularly *Ae. koreicus* in 2011 [33] and *Ae. japonicus* in 2015 [34], along with the local transmissions that have occurred, have highlighted the importance of maintaining strong entomological surveillance systems capable of detecting changes in mosquito populations and identifying newly introduced species. In response, national and regional authorities have implemented entomological surveillance programmes aimed at monitoring mosquito populations and improving early detection capacity [35]. In 2020, the Italian National Plan for Prevention, Surveillance and Response to Arbovirus 2020–2025 (PNA 2020–2025) [35] was updated, with the aim of enhancing the surveillance and control of AIM vectors and re-emerging vector-borne diseases at the national level, including through strengthening the monitoring and early detection of invasive *Aedes* species at PoEs.

Despite the recognised importance of transport hubs in vector introductions, relatively few long-term studies on surveillance programmes have specifically focused on PoEs in Europe [36,37,38,39,40]. Nevertheless, monitoring activities conducted at major airports and ports have demonstrated that these locations may act as gateways for the introduction of AIMs through international travel and trade [37,38]. The identification of newly introduced species at PoEs may therefore represent an early warning signal for the potential establishment and subsequent spread of mosquito populations in surrounding areas.

Within this framework, an entomological surveillance programme targeting AIMs at PoEs was established in 2018 in the Veneto region, northeastern Italy and has been conducted continuously since then. The present study reports the results of surveillance activities carried out between 2018 and 2024 during the mosquito season (June–October). Specifically, the programme aims to strengthen surveillance at major transportation hubs by (i) monitoring mosquito species composition and (ii) assessing temporal changes in the population dynamics of major vector species, thereby supporting the prevention of both the introduction and outward spread of AIMs through passive transport associated with passenger and cargo traffic.

## 2. Materials and Methods

### 2.1. Study Area

The study area includes three monitoring stations located at Treviso Airport, Venice Airport, and Venice Port in the Veneto region. These sites represent key transport routes in northeastern Italy and serve as major Italian hubs for both passenger and cargo traffic in the maritime and air transport sectors. Treviso-Sant’Angelo Airport “Antonio Canova” is a relatively small airport, which serves as an important gateway for tourism to Venice and other cities of northern Italy due to its strategic connections to European countries. The Venice International Airport “Marco Polo”, located in Tessera, ranks among the busiest airports in the country, with over 1000 weekly connections to major national, European, and intercontinental destinations. In 2024, the airport handled more than 11 million passengers, primarily from other Italian regions and European countries (e.g., Germany, France, and Spain). Additionally, the main cargo routes included connections with Germany, North America (e.g., New York, Philadelphia, Chicago, Atlanta, Montreal), and the Middle East (e.g., Istanbul, Dubai, Qatar), with a total cargo throughput of 54,731.3 tons. The port of Venice, located in Marghera, is one of the largest coastal industrial areas in Europe and one of Italy’s main ports in terms of commercial traffic, owing to the well-developed transport infrastructure and its industrial, commercial, and service activities.

All sites are characterized by a highly anthropized landscape, with large-scale built infrastructure (e.g., terminals, hangars, and control towers) and extensive cemented areas (e.g., runways, aprons, and artificial docks). Green spaces are confined to isolated fragments/marginal grass strips and limited water sources, mainly represented by catch basins, storm drains, and small temporary water accumulations directly linked to rainfall events, offering typically larval habitat for *Aedes* species.

The three monitored sites were subject to periodic treatments for mosquito control according to current regulations [28,35,41,42]. Interventions targeting both transport vehicles (aircraft and ships) and infrastructural areas, including terminals and the external perimeters of airports and port, were implemented to prevent mosquito infestation. Specifically, larvicides Diflubenzuron or Pyriproxyfen (insect growth regulators) and *Bacillus thuringiensis israelensis* (Bti), as well as pyrethroid-based adulticides (e.g., Cypermethrin and Deltamethrin), were applied with a rotation of active substances in both airside and landside terminals and their external perimeters. Adulticide treatments were performed monthly between May and September, while larvicides were generally applied from April to October according to the instructions of the specific product (e.g., 2–4 week intervals).

### 2.2. Mosquito Sampling

**Figure 1:**
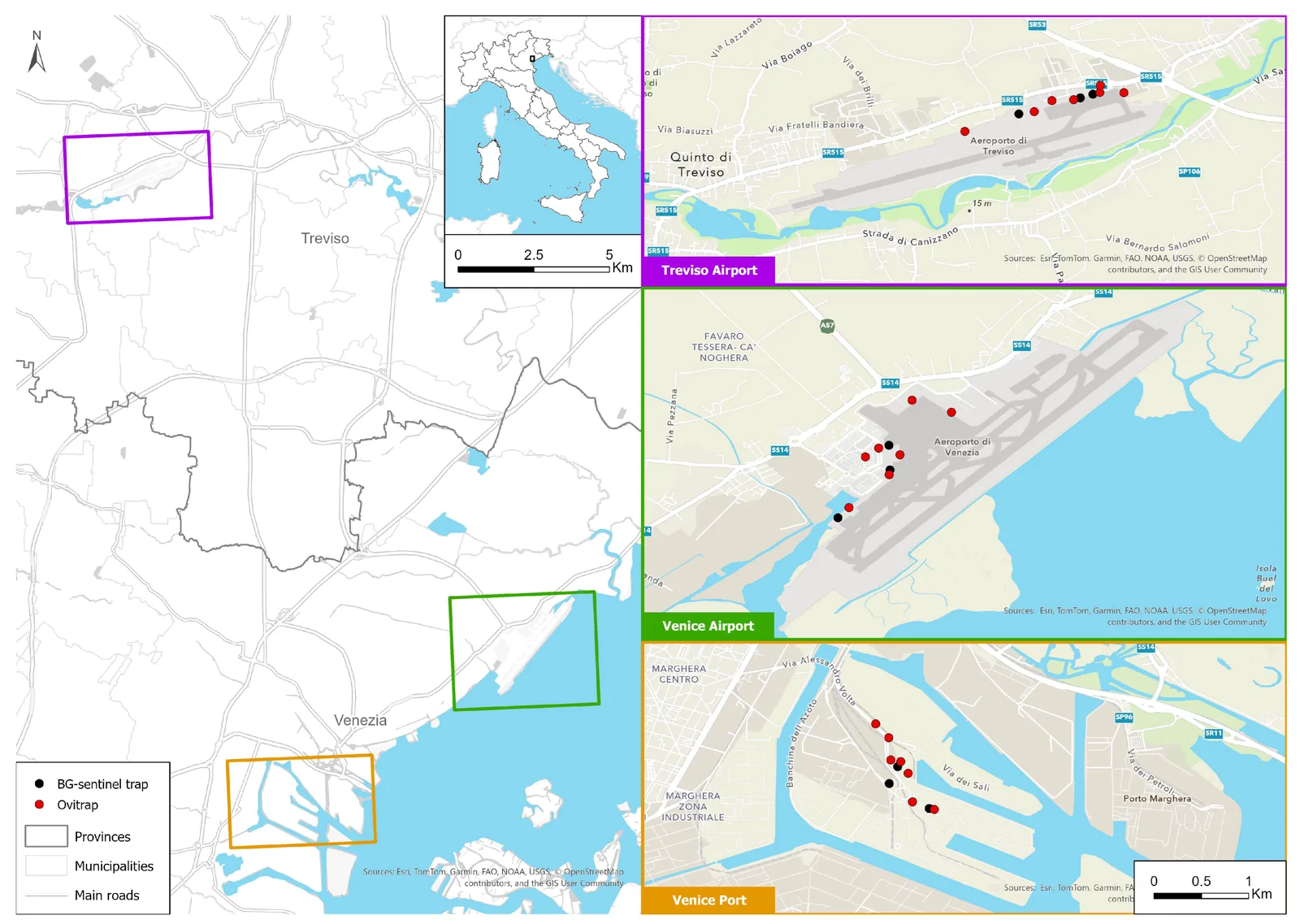
Map of the Points of Entry included in the entomological monitoring conducted from 2019 to 2024 in northeastern Italy (Veneto region). The left panel shows the three monitored sites: Treviso Airport (purple rectangle), Venice Airport (green rectangle), and Venice Port (orange rectangle). The right panel provides an enlarged view of the monitored areas, showing the georeferenced trap locations. Black dots indicate the positions of the three BG-Sentinel traps, whereas red dots indicate the positions of the seven ovitraps deployed at each site.

Within each airport and the port, different traps were positioned in key areas for mosquito introduction and infestation, including passenger waiting areas, vegetated zones (near parking and airport buildings), cargo terminals, and baggage handling areas. The surveillance programme (2018–2024) consisted of a pilot survey, followed by a long-term entomological monitoring conducted annually during the seasonal peak of mosquito activity. The pilot survey was carried out in 2018 deploying ten ovitraps per site from early July to early October to identify primary hotspot areas for mosquitoes. Starting in 2019, each airport and the port was monitored using seven ovitraps and three BG-Sentinel traps (BGS; Biogents AG, Regensburg, Germany) operating continuously from late June to early October until 2024 (Figure 1, Supplementary File S1). These trap types were selected for their specificity in collecting *Aedes* eggs and adults, respectively. The ovitraps consisted of 400 mL black plastic containers filled with 300 mL of water and equipped with a masonite^TM^ stick (3 cm width × 15 cm length) for egg deposition, whereas BGS traps were baited with BG-Lure (Biogents AG, Regensburg, Germany) and connected continuously to the power supply. Masonite sticks and water in the ovitraps, as well as catch bags containing adult mosquitoes, were collected and replaced weekly with fresh sampling materials.

### 2.3. Morphological and Molecular Mosquito Identification

Throughout each year of monitoring, all mosquito eggs and adult specimens were counted and identified to define number, sex, and species of mosquitoes at each location. Species identification from egg samples was performed following hatching and subsequent development to at least the fourth instar larvae or until adult emergence. Mosquito rearing was carried out at the insectary of the Istituto Zooprofilattico Sperimentale delle Venezie under controlled conditions (temperature: 27 ± 2 °C; relative humidity: 80 ± 5%; light-dark: 16–8 h). Morphological species-specific characters were evaluated to identify both larvae and adult mosquitoes using a stereomicroscope and standard taxonomic keys [30,43,44]. In cases of ambiguous morphological identification or suspected introduction of a new species, identification was confirmed by molecular analysis. This approach was applied to confirm uncertain morphological identifications of *Ae. koreicus*/*Ae. japonicus* specimens, but was not used for *Ae. albopictus*. Specifically, individual specimens were first homogenized using two 5 mm tungsten steel beads in a TissueLyser II (QIAGEN, Valencia, CA, USA) at 30 Hz for two 30 s rounds, to facilitate tissue disruption. DNA was extracted from homogenate with an automatic extractor (Microlab STAR Hamilton, Americas, Australia & Pacific Rim and KingFisher Apex, Thermo Fisher Scientific, Waltham, MA, USA) using MagMAX Pathogen RNA/DNA kit (Thermo Fisher Scientific) following the high-volume manufacturer protocol.

DNA was amplified from each specimen at two mitochondrial loci: nicotinamide adenine dinucleotide dehydrogenase subunit 4 (ND4) and cytochrome oxidase I (COI).

For ND4, PCR reactions were performed using the primer pair N4J-8502D and N4N-8944D (N4J-8502D: 5′-CGTAGGAGGAGCAGCTATATT-3′; N4N-8944D: 5′-AAGGCTCATGTTGAAGCTCC-3′) targeting a ~465 bp long fragment [45]. The final volume of 20 μL contained 10 μL of 2x QuantiFast SYBR Green PCR Master Mix (Qiagen, Hilden, Germany), 0.3 μM of each primer and 3 μL of DNA template. The cycling conditions involved a denaturation of 95 °C for 5 min, followed by 40 cycles at 95 °C for 15 s, 55 °C for 30 s and 60 °C for 30 s, with a final dissociation step of 95 °C for 15 s, 60 °C for 1 min and a melt ramp up to 95 °C with 0.3 °C/s increments.

Additionally, partial COI sequences (~710 bp) were obtained using the universal primer pairs LCO1490 and HCO2198 (LCO1490: 5′-GGTCAACAAATCATAAAGATATTGG-3′; HCO2198: 5′-TAAACTTCAGGGTGACCAAAAAATCA-3′) [46] to confirm the species identification results and conduct a phylogenetic analysis. The PCR reaction was carried out in a total volume of 50 μL, containing 2 units of AmpliTaq Gold DNA Polymerase (Applied Biosystem, Thermo Fisher Scientific), 5 μL of 10X PCR Buffer, 2 mM of MgCl2, 0.2 mM of each dNTP, 0.4 μM of primers LCO1490-HCO2198, and 5 μL of extracted DNA. The thermal profile consisted of activation at 95 °C for 10 min, followed by 35 cycles pf denaturation at 95 °C for 30s, annealing at 55 °C for 30 s, and extension at 72 °C for 30 s, with a final extension step of 7 min at 72 °C.

The PCR products obtained for both loci (ND4 and COI) were sequenced bidirectionally using the corresponding reverse and forward primers. The resulting electropherograms were inspected using the FinchTV chromatogram viewer (Version 1.4.0) and consensus sequences were assembled using MEGA11 (Version 11.0.13) [47].

All the *Aedes koreicus* ND4 and COI sequences were compared with most similar sequences identified through BLAST (https://blast.ncbi.nlm.nih.gov/Blast.cgi, accessed on 25 August 2026), to confirm species identification [48].

All sequences generated in this study were deposited in GenBank under accession numbers PZ863445–PZ863448 for ND4 gene and PZ869459–PZ869460 for COI gene.

### 2.4. Phylogenetic and Haplotype Network Analyses

COI sequences were selected for the phylogenetic analyses because this marker is more widely used for this purpose in the literature than ND4, thereby allowing the inclusion of a larger number of reference sequences for comparison. Accordingly, COI sequences were obtained from two of the four *Ae. koreicus* specimens captured during the PoE surveillance programme (specimens from Venice Airport, 2018, and one of the two from Treviso Airport, 2019, accession numbers: PZ869459 and PZ869460, respectively) and were analysed to assess their genetic relationships with other *Ae. koreicus* sequences available in GenBank.

For the phylogenetic analysis, the dataset was assembled by querying the COI sequences obtained in this study against the NCBI GenBank database using BLAST [48]. All available COI sequences of *Aedes koreicus* were retrieved, together with five *Aedes japonicus* sequences, which were included as an outgroup. The final dataset consisted of 273 retrieved through BLAST in addition to the two sequences generated in this study. Sequences were annotated using metadata obtained from GenBank records and the published literature, including the country and year of collection, which were incorporated into the sequence names. The dataset was subsampled by removing identical sequences sharing the same country and date of collection and retaining a single representative sequence from each group. Maximum-likelihood phylogenetic inference was performed using IQ-TREE v3.0.1, and node support was assessed with 100 non-parametric bootstrap replicates [49,50]. Pairwise genetic distances (p-distances) were calculated among all sequences and within and between the two major clades identified in the phylogenetic analysis, using Mega11 [47].

For the haplotype network reconstruction, the full dataset, including all available *Ae. koreicus* COI sequences, was converted to NEXUS format using MEGA11 [47]. Sequences containing more than 10 degenerate bases were removed from the dataset. The resulting NEXUS file was manually edited by manually adding the geographic traits section. The file was imported into PopART, and a haplotype network was constructed using the median-joining algorithm (ε = 0) [51].

### 2.5. Statistical Analysis

To investigate the temporal dynamics of *Ae. albopictus* population, egg (from ovitraps) and adult counts (from BGS) were analysed separately using a Negative Binomial Regression (Type 1 for egg counts and Type 2 for adults) via the *glmmTMB* package in R version 1.1.11 [52]. To reduce spatial autocorrelation arising from fixed trap position, the response variables (weekly mosquito egg or adult counts data) were weekly aggregated by site (i.e., Treviso Airport, Venice Airport, Venice Port). In both models, the year (i.e., 2019–2024) and the week (i.e., 25–41) of collection were included as categorical fixed effects, while the variable site was treated as random effect to account for unmeasured environmental heterogeneity. To account for differences in sampling effort (i.e., different number of active traps per each collection) an offset term defined as the natural logarithm of the number of active traps was included in the model. The model outputs are presented as Estimated Marginal Means (EMMs) with their corresponding 95% Confidence Intervals (95% CI) back-transformed from the model’s log-scale to the original response scale (abundance counts) for biological interpretation. Both EMMs and their 95% CIs were calculated using the *emmeans* package version 2.0.2 [53] to obtain unbiased population-level predictions (both weekly and annual) by controlling confounding effects of site-specific differences and unequal sampling effort. Model assumptions were validated using simulated randomized quantile residuals via the *DHARMa* package version 0.4.7 [54] to detect and verify (i) heteroscedasticity and nonlinearity of data, (ii) overdispersion and deviation from expected distribution, and (iii) temporal independence of residuals via the Durbin–Watson test.

Environmental data were obtained from the IZSVe EVE dataset [55] and extracted for the three study areas. Specifically, land surface temperature (LST) data were derived from MODIS satellite imagery and converted to degrees Celsius, whereas precipitation data were obtained by spatial interpolation of rainfall measurements recorded by ARPAV meteorological stations and expressed in millimetres (mm) [56].

## 3. Results

### 3.1. Mosquito Species Identification

During the pilot survey in 2018, out of the 24,522 (Treviso Airport = 10,862; Venice Airport = 8305; Venice Port = 5355) *Aedes* eggs collected, one *Ae. koreicus* larva hatched from samples collected at Venice airport. The remaining eggs collected from each sampling site were all identified as *Ae. albopictus* specimens.

During the following entomological monitoring conducted from 2019 to 2024, a total of 71,671 eggs (Treviso Airport = 28,712; Venice Airport = 22,770; Venice Port = 20,189) were collected. Based on the hatched larvae, all eggs were identified as belonging to the *Ae. albopictus* species, with the occasional finding of some *Culex pipiens* egg rafts (excluded from the count). Concerning adult mosquitoes, 27,142 specimens were collected, of which 99.5% were identified to species level. Over the years, 12 species were identified as reported in Table 1. Among the AIMs, *Ae. albopictus* was the dominant species in the study area and represented 52.4% (N = 14,231) of the total sample, consistent with its widespread establishment across Italy. Additionally, two adults of *Ae. koreicus* were also reported at Treviso Airport in 2019 and one in Venice Airport in 2020. This species has a more limited distribution and occurs at lower densities than *Ae. albopictus* [57]. The remaining species collected were native mosquito species commonly found in the Italian mosquito fauna.

### 3.2. Interannual and Seasonal Dynamics of Aedes albopictus Population

**Figure 2:**
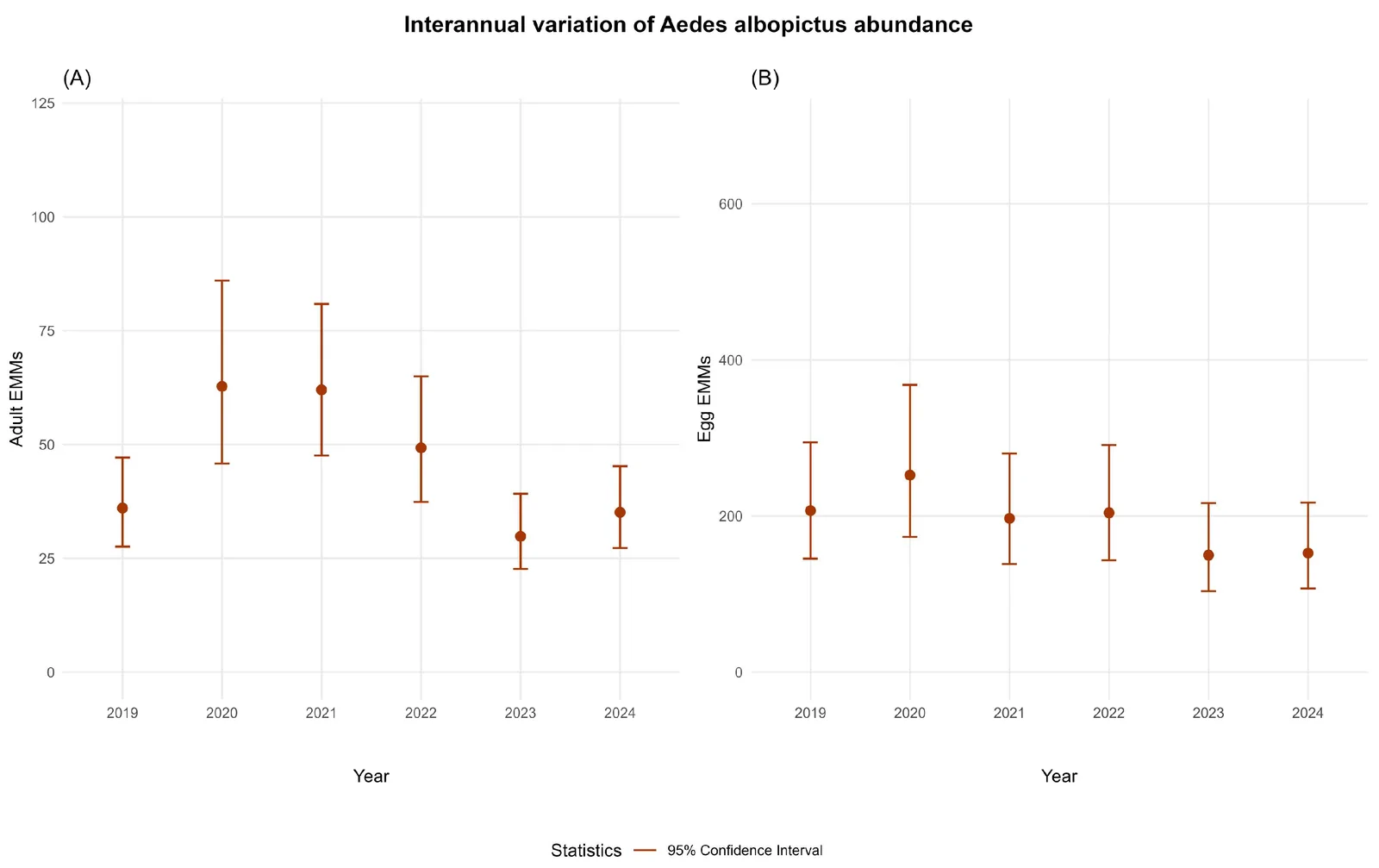
Interannual variation of *Aedes albopictus* abundance. Model outputs were visualised as Estimated Marginal Means (EMMs) with their corresponding 95% Confidence Intervals (95% CI) of adult (**A**) and egg (**B**) counts averaged at the level of week.

The interannual monitoring (2019–2024) of *Ae. albopictus* population revealed highly consistent temporal patterns between the two sampling methods applied to collect egg (ovitraps) and adult (BGS) mosquitoes (Figure 2). Both eggs and adult population were characterized by an initial higher density period followed by lower abundance.

Within the high-density window, adult abundances peaked during 2020 (EMM = 62.8, 95% CI = 45.8–86.0) and 2021 (EMM = 62.0, 95% CI = 47.6–80.8) followed by 2022 (EMM = 49.3, 95% CI = 37.4–65.0) and 2019 (EMM = 36.0, 95% CI = 27.6–47.1) (Figure 2A). Similarly, ovitrap collections exhibited a stable population abundance throughout the 2019–2022 period, with the peak in abundance recorded in 2020 (EMM = 252.6, 95% CI = 173.2–368.4) and high values reported across these years. Conversely, a drop occurred during the 2023–2024 period, with the lowest overall abundance recorded in 2023 (egg EMM = 149.9, 95% CI = 103.8–216.6; adult EMM = 29.8, 95% CI = 22.7–39.2) followed by 2024 (egg EMM = 152.6, 95% CI = 107.2–217.2; adult EMM = 35.1, 95% CI = 27.2–45.2) (Figure 2B).

**Figure 3:**
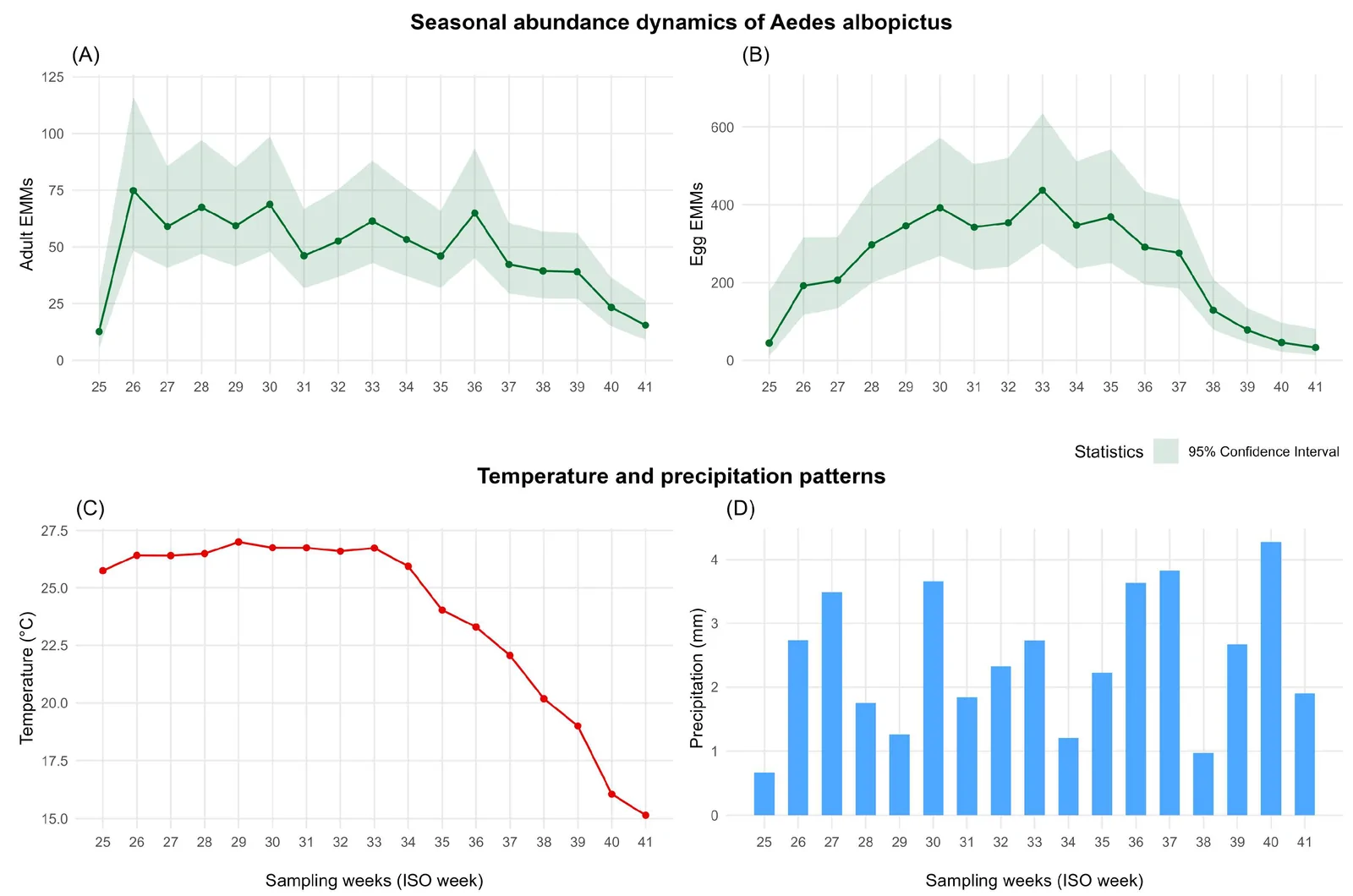
Seasonal abundance dynamics of *Aedes albopictus* with temperature and precipitation patterns. The top panels represent model outputs, visualised as Estimated Marginal Means (EMMs) with their corresponding 95% Confidence Intervals (95% CI) of adult (**A**) and egg (**B**) counts. The bottom panels present the observed weekly mean temperature (**C**) and precipitation (**D**) across the seasonal monitoring.

Regarding seasonal dynamics, although the temporal distribution of observations differed among years, adult abundance remained relatively stable throughout the activity season, without a clearly defined seasonal peak. A low density was reported in early summer (Week 25), with an EMM of 12.7 adult mosquitoes (95% CI = 5.3–30.5) per BGS trap (Figure 3A) and 46.0 eggs per ovitrap (95% CI = 11.5–184.8) (Figure 3B). Thereafter, a sharp and significant increase in adult (EMM = 74.9; 95% CI = 48.2–116.2) and egg abundance (EMM = 191.9; 95% CI = 116.4–316.4) started in late June (Week 26), followed by a stable high population density throughout the core summer season. Our analysis indicated that egg abundance reached its maximum during mid-August (Week 33 EMM = 437.6, 95% CI = 301.6–634.8) with slight and not significant decreases reported within the summer weeks (27–37). In early autumn, marked differences in population dynamics were observed with a sharp and highly significant population decline established early for eggs, followed by a slower decrease of adult specimens. Indeed, the drop in oviposition started at Week 38 (EMM = 128.7, 95% CI = 79.2–209.1), reaching its minimum by Week 41 (EMM = 32.9, 95% CI = 13.4–80.6). Conversely, the adult abundance exhibited a gradual reduction, with low values matching early-season levels only at the end of monitoring (EMM = 15.5, 95% CI = 9.1–26.4).

The warmest weeks throughout the study area are reported between weeks 26 and 33 (from late June to mid-August), with mean temperatures ranging from 26.4 to 27 °C. Precipitation patterns exhibit both interannual and seasonal variability, with the lowest mean value of 0.7 mm recorded during week 25 and the highest mean precipitation of 4.1 mm in early autumn, during week 40.

### 3.3. Phylogenetic Analysis

**Figure 4:**
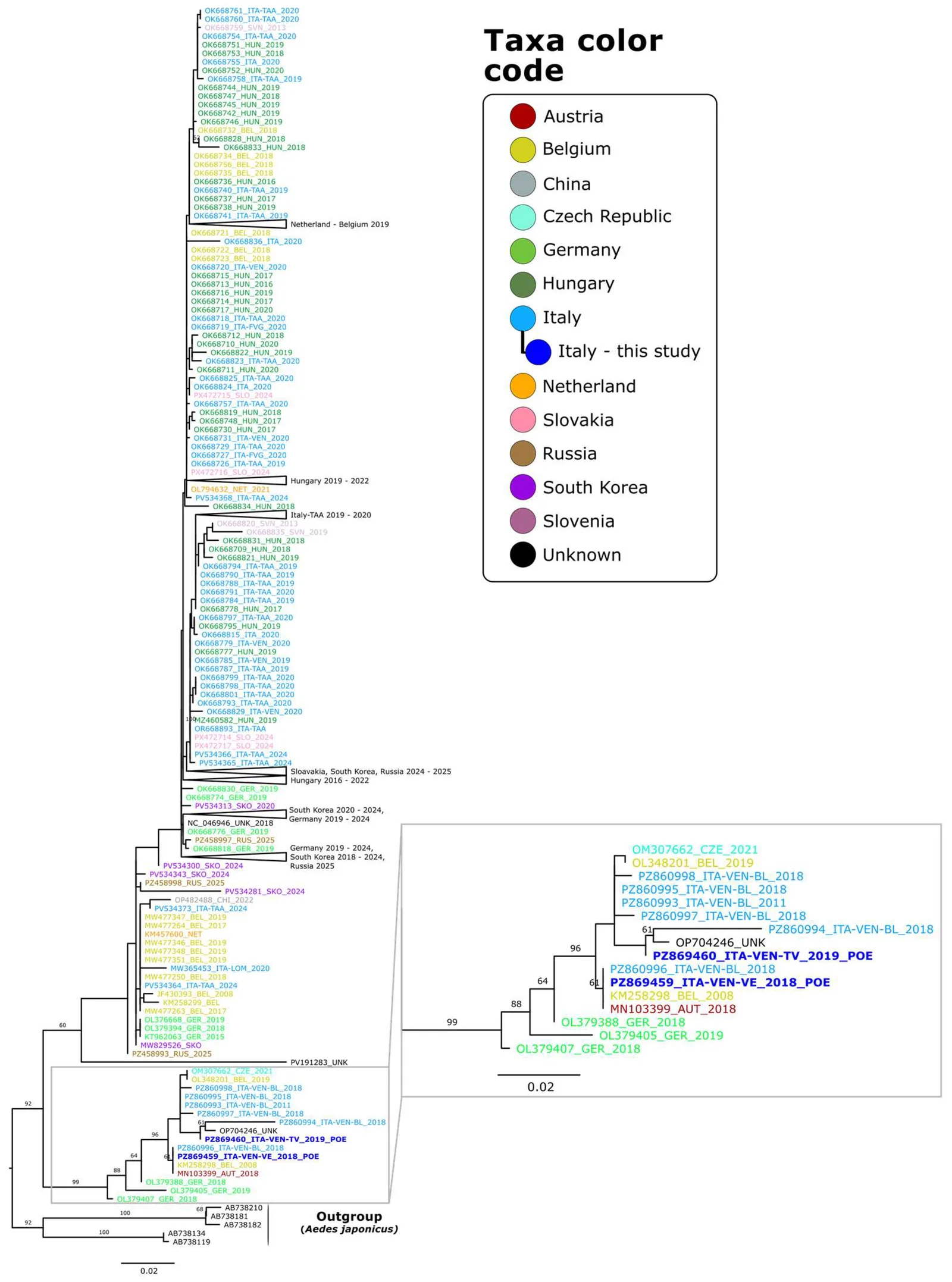
Maximum-likelihood phylogenetic tree inferred from partial COI sequences of *Aedes koreicus*. Node support values (non-parametric bootstrap) are shown at the nodes when ≥60. Tips are colour-coded according to country of origin. Sample codes refer to country and year of collection for European samples, and to country, region, province, and year of collection for Italian samples included in this study (FVG = Friuli-Venezia Giulia, LOM = Lombardy, TAA = Trentino-Alto Adige, VEN = Veneto). The inset shows a detailed view of the clade in which the samples collected at Points of Entry cluster.

The phylogenetic analysis of the partial COI sequences reveals the presence of two distinct clades within the *Ae. koreicus* species (Figure 4). The main clade includes most of the publicly available sequences and comprises sequences from Europe and Asia. The smaller clade includes only a few sequences obtained from European samples. The samples collected at Venice and Treviso Airports in 2018 and 2019 (PZ869459_ITA-VEN-VE_2018_POE and PZ869460_ITA-VEN-TV_2019_POE, respectively) clustered within the smaller clade, which also included samples collected in Italy (six sequences from the same geographical area), Austria, Belgium, the Czech Republic, and Germany between 2008 and 2021. The oldest Italian sample within this clade was collected in Belluno (Veneto region) in 2011, representing the first record of *Ae. koreicus* in Italy. Notably, this clade is well supported and clearly separated from the remaining *Ae. koreicus* sequences collected across Europe, Russia, South Korea, and China. Within this small clade the samples PZ869459_ITA-VEN-VE_2018_POE and PZ869460_ITA-VEN-TV_2019_POE display the highest genetic similarity (100%) with two samples from Belluno province collected in 2011 and 2018, a sample from Belgium (2008) and one from Austria (2018). The mean p-distance between the two *Ae. koreicus* clades was 5.56%, whereas the mean within-clade p-distances were 0.63% and 1.01% for the larger and smaller clades, respectively (Supplementary File S2).

### 3.4. Haplotype Network Analysis

**Figure 5:**
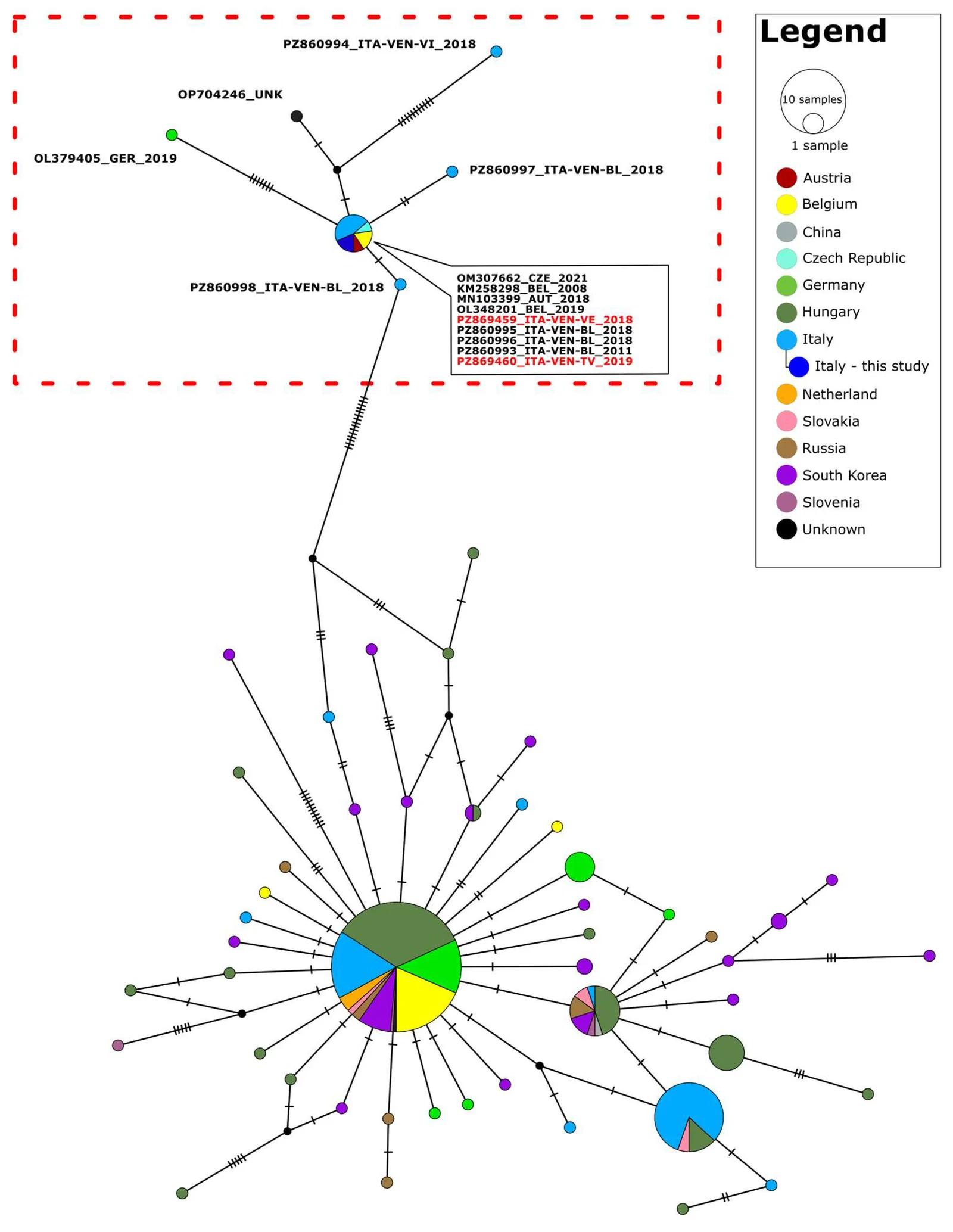
Haplotype network based on available COI sequences. Colours indicate the country of origin of each sample; Italian samples generated in this study are shown in dark blue. Black nodes represent inferred intermediate haplotypes not detected in the dataset. Hatch marks indicate the number of mutational steps between haplotypes. The red dotted line highlights the newly identified European population of *Aedes koreicus*.

The haplotype network (Figure 5) further supports the result obtained in the phylogenetic analysis. The COI haplotype network was characterised by a dominant haplotype widely distributed across Europe and Asia, to which most sequences belonged. The clade identified in the phylogenetic analysis is clearly separated also in the haplotype network, highlighting a potential different population.

## 4. Discussion

International guidelines recognise surveillance at PoEs as a key component of integrated vector management, enabling the early detection of AIMs and timely control measures. Indeed, PoEs represent critical gateways for the introduction and spread of AIMs, as the increasing international movements of passengers, goods and vehicles continuously raises the probability of passive vector transport. In this study, we present a comprehensive multi-year assessment (2018–2024) of mosquito populations at three major PoEs (Treviso Airport, Venice Airport and Venice Port) in northeastern Italy, highlighting the value of long-term entomological surveillance. Indeed, short-term surveys may fail to detect rare or newly introduced species, particularly when mosquito populations are still small or localized. In contrast, long-term monitoring increases the probability of detecting introduction events and provides valuable insights into mosquito community composition and the temporal dynamics of established vector populations, as also demonstrated by long-term PoE surveillance programmes conducted in other European countries [36,37,38,40]. However, sustaining long-term surveillance at PoEs may pose operational challenges, particularly regarding stable funding, trained personnel and coordination among stakeholders. Integrating surveillance activities into existing vector control and public health programmes, together with a risk-based prioritization of sites and standardized protocols, may help optimize resources and ensure the continuity and sustainability of surveillance over time.

In this study, although no new AIM introductions were recorded, the detection of *Ae. koreicus* at both Treviso Airport (2019) and Venice Airport (2018 and 2020) represents an important outcome of the surveillance programme. In fact, these findings highlight the expansion of this invasive species in northern Italy [13,57,58] and the risk of its exportation to other areas/countries through transport hubs. Specifically, the occurrence and spread in the Province of Treviso had already been reported since 2012, reaching 28 positive municipalities out of 94 (29.8%) by 2019, which strongly supports dispersal from surrounding areas [13]. The specimen collected at Venice Airport in 2018 represents the first documented detection of *Ae. koreicus* in this coastal area, highlighting an unusual occurrence compared with its previously known Italian distribution, which was mainly associated with high-elevation inland areas (typically at 400–500 m a.s.l.) characterized by mild summer temperatures and higher precipitation [57,59,60,61,62]. However, its altitudinal range appears broader than that of other AIMs and its relatively recent invasion history may explain its absence or limited occurrence in some areas. Considering the geographic distribution and environmental traits of previously colonised sites in Italy, the introduction of *Ae. koreicus* at Venice Airport can likely be attributed to passive human-mediated transport through road networks, highlighting the role of transportation hubs in invasive mosquito dispersal beyond their typical ecological settings. Climate warming may further contribute to the expansion of the species’ distribution ranges by extending the seasonal window of temperatures suitable for its development and reproduction (23–28 °C), potentially advancing the onset and delaying the end of the breeding season [63].

Phylogenetic and haplotype network analyses based on COI sequences revealed that the *Ae. koreicus* specimens collected at Venice airport (2018) and at Treviso Airport (2019) were closely related to specimens collected in the Veneto region in 2011 (first finding) and in 2018 (sequences available in GenBank). These specimens clustered within a distinct clade that was clearly separated from the majority of the European *Ae. koreicus* COI sequences currently available in public databases. Notably, the specimens collected at Venice Airport in 2018 and at Treviso Airport in 2019 clustered together with the first Italian sequences (2011), other Veneto sequences (2018), and sequences from other European countries, including Austria and Belgium, whereas other Italian sequences from Veneto and Trentino clustered within a separate clade. The occurrence of genetically differentiated *Ae. koreicus* lineages among Italian specimens collected within a relatively short time interval may provide insights into the possible invasion history of the species in Europe. This genetic variation could have been present in the source populations before introduction into Italy, rather than having arisen through recent differentiation following establishment. Thus, the observed pattern is compatible with repeated introduction events involving genetically differentiated source populations and potentially different invasion pathways. However, this interpretation should be considered as a hypothesis rather than definitive evidence of multiple introductions. COI represents only a fragment of a single mitochondrial gene, and the observed clustering cannot distinguish between independent introduction events and other alternative demographic or evolutionary scenarios. Concordant patterns based on multiple independent genetic markers, ideally including genome-wide data from both native and introduced populations, would provide stronger evidence for reconstructing the number and geographic origins of introduction events. Nevertheless, the detection of genetically distinct lineages among Italian populations highlights the genetic diversity of *Ae. koreicus* in Europe and supports the possibility of repeated transport-mediated introductions. Given the co-circulation of different genetic variants in Europe and Italy and the genetic distance between the Venice specimen and most publicly available sequences, these findings are consistent with previous studies [64,65]. Overall, these results suggest a complex invasion scenario, in which an independent dispersal pathway from the native Asian range or a secondary introduction via other European countries cannot be ruled out. However, the limited availability of sequences from the native range and the use of a single mitochondrial marker prevent definitive conclusions regarding the origin and invasion pathway of this population. Broader genomic analyses based on whole-genome sequencing, including multiple native and non-native populations, will be essential to investigate the geographic origins of this species in Italy, as well as in other parts of Europe.

Regarding the species collected during this surveillance programme at the POEs, the relative abundances observed should not be considered representative of the actual mosquito communities at the monitored sites, as the traps used were specifically designed for the surveillance of AIMs. Among all collected species, *Ae. albopictus* was the most common mosquito at all monitored sites and also the dominant species at both Venice and Treviso airports. The remarkable ecological plasticity of *Ae. albopictus*, together with its long-term invasion history [32,66], largely explains its widespread presence in the study area, as in other parts of Italy. The presence of stable populations at relatively high densities within these transportation hubs is particularly relevant, as they may substantially increase the likelihood of vector exportation to areas where the species is not yet established. These findings also imply that the routine control interventions implemented at these sites may have limited effectiveness on *Ae. albopictus* populations, highlighting the difficulty of eliminating the species once established. Notably, high reproductive activity and sustained adult density were reported throughout the summer season, with egg abundance exceeding the epidemiological threshold reported during the 2007 chikungunya outbreak in Italy (44.15 eggs/ovitrap/week) [67]. These results warrant attention and highlight the need to integrate continuous surveillance with vector control programmes and to establish evidence-based action thresholds calibrated on specific environmental conditions and historical entomological data to optimize treatment timing and guide targeted interventions [68].

Beyond the risk of vector introduction into new territories, mosquito surveillance at these sites may play a key role in detecting increasing vector pressure and emerging risk of pathogen transmission also in surrounding areas. Indeed, the high *Ae. albopictus* egg and adult densities recorded in 2020 might have represented a warning signal, considering their coincidence with the first autochthonous dengue outbreak reported in Italy (between July and August), which occurred in Vicenza Province, Veneto Region. Due to a prompt response, including intensive vector control and both active entomological and human surveillance in the affected area, the outbreak was successfully contained among household contacts of the index case [25]. While our data do not establish a causal relationship between vector abundance at the monitored sites and the occurrence of local virus transmission events, they support the concept that significant increases in mosquito abundance may represent an early warning signal deserving particular attention within integrated regional surveillance systems, specifically in sites where vector control activities are implemented.

Finally, several non-mutually exclusive factors could explain the observed densities, as well as seasonal and interannual trends, including climatic conditions, operational differences in control interventions, or a progressive reduction in insecticide efficacy resulting from resistance mechanisms. Distinguishing among these potential drivers was beyond the scope of the present study but should represent a priority for future research. Future studies integrating entomological data, climatic variables and detailed control intervention will be necessary to determine the effects of these variables on population size, establish action thresholds and define whether mosquito control programmes could be improved to effectively reduce mosquito populations, achieve maximum cost-effective benefit from control interventions and prevent both mosquito introduction and vector-borne disease transmission.

## 5. Conclusions

Overall, this long-term surveillance study confirms the key role of PoEs as strategic sites for the early detection of AIMs and the monitoring of established vector populations. Although no new introductions were recorded, the detection of *Ae. koreicus* at Venice and Treviso airports highlights the ongoing expansion of this invasive species in northern Italy and the potential contribution of transport hubs to its dispersal. The persistent presence and high seasonal abundance of *Ae. albopictus* further demonstrate the importance of maintaining surveillance programmes capable of detecting changes in mosquito population dynamics and providing early warning signals of increased vector pressure. Integrating entomological surveillance with climatic, operational, and resistance data will be essential to optimize control strategies, improve understanding of invasion pathways, and reduce the risk of AIM establishment and vector-borne disease transmission.

## Figures and Tables

**Table 1:** Number of adult mosquitoes collected by BGS with lure at each sampling site (Treviso Airport, Venice Airport and Venice Port) and in total, reported by mosquito species. The asterisk indicates species that are invasive in Italy (AIMs).

Species (Adults)	Treviso Airport	Venice Airport	Venice Port	Total
*Ae. albopictus* *	6019	4120	4092	14,231
*Ae. caspius*	26	279	184	489
*Ae. koreicus* *	2	1	0	3
*Ae. vexans*	3	5	0	8
*An. claviger*	1	1	0	2
*An. maculipennis s.l.*	20	3	2	25
*Cs. annulata*	0	0	1	1
*Cs. longiareolata*	1	0	3	4
*Cx. hortensis*	8	0	0	8
*Cx. modestus*	3	0	0	3
*Cx. pipiens*	1695	1169	9378	12,242
*Cx. territans*	0	0	1	1
*Anopheles* sp.	2	0	0	2
Not identified	8	77	38	123
Total	7788	5655	13,699	27,142
* AIMs				

## Data Availability

The original contributions presented in this study are included in the article/Supplementary Material. Further inquiries can be directed to the corresponding author(s).

## Supplementary Materials

The following supporting information can be downloaded at https://www.mdpi.com/article/10.3390/tropicalmed11090247/s1, Supplementary File S1. Dates of trap placement and collection and corresponding collection weeks (ISO week). Supplementary File S2. Table S1. Estimates of evolutionary divergence between sequences. The number of base differences per site from between sequences are shown. Standard error estimate(s) are shown above the diagonal. This analysis involved 239 nucleotide sequences. Codon positions included were 1st + 2nd + 3rd + Noncoding. All ambiguous positions were removed for each sequence pair (pairwise deletion option). There were a total of 1529 positions in the final dataset. Evolutionary analyses were conducted in MEGA11 [47]. Table S2. Estimates of evolutionary divergence over sequence pairs between groups. The number of base differences per site from averaging over all sequence pairs between groups are shown. Standard error estimate(s) are shown above the diagonal. This analysis involved 239 nucleotide sequences. Codon positions included were 1st + 2nd + 3rd + Noncoding. All ambiguous positions were removed for each sequence pair (pairwise deletion option). There were a total of 1529 positions in the final dataset. Evolutionary analyses were conducted in MEGA11 [47]. Table S3. Estimates of average evolutionary divergence over sequence pairs within groups. The number of base differences per site from averaging over all sequence pairs within each group are shown. Standard error estimate(s) are shown in the second column. This analysis involved 239 nucleotide sequences. Codon positions included were 1st + 2nd + 3rd + Noncoding. All ambiguous positions were removed for each sequence pair (pairwise deletion option). There were a total of 1529 positions in the final dataset. Evolutionary analyses were conducted in MEGA11 [47].

## Abbreviations

The following abbreviations are used in this manuscript:

AIMs	*Aedes* Invasive Mosquitoes
DENV	Dengue Virus
CHIKV	Chikungunya Virus
ZIKV	Zika Virus
PoEs	Points of Entry
IVM	Integrated Vector Management
ECDC	European Centre for Disease Prevention and Control
Bti	*Bacillus thuringiensis israelensis*
BGS	BG-Sentinel trap
EMM	Estimated Marginal Mean
CI	Confidence Interval
